# Prophylactic Protective Effects and Its Potential Mechanisms of IcarisideII on Streptozotocin Induced Spermatogenic Dysfunction

**DOI:** 10.3390/ijms150916100

**Published:** 2014-09-11

**Authors:** Yongde Xu, Hongen Lei, Ruili Guan, Zhezhu Gao, Huixi Li, Lin Wang, Yu Hui, Feng Zhou, Zhongcheng Xin

**Affiliations:** 1 Molecular Biology Laboratory of Andrology Center, Peking University First Hospital, Peking University, Beijing 100034, China; E-Mails: xuyongd@foxmail.com (Y.X.); hongenlei@foxmail.com (H.Lei); guanruili@gmail.com (R.G.); gaozhezhu@gmail.com (Z.G.); huixilee@gmail.com (H.Li); texutijian@163.com (L.W.); 2 Department of Urology, First Affiliated Hospital of Soochow University, Soochow University, Suzhou 215006, China; E-Mails: huiyfishing@126.com (Y.H.); always301@sina.com (F.Z.)

**Keywords:** streptozotocin, spermatogenic dysfunction, IcarisideII, oxidative stress, diabetes mellitus

## Abstract

The aim of this study was to investigate the effects of IcarisideII(ICAII) on the prevention of streptozotocin (STZ) induced spermatogenic dysfunction. Forty male Sprague-Dawley rats received intraperitoneal injection of STZ (55 mg/kg) and were equally randomized to gavage feeding of vehicle (the vehicle group) or ICAII (0.5, 1.5 or 4.5 mg/kg/day, respectively). Ten normal rats received vehicle and served as control. Four weeks later, sperm parameters, histopathological changes, testicular lipid peroxidation, antioxidant enzyme activities, and apoptosis index (AI) were evaluated. Results showed that ICAII treatment resulted in a significant recovery of sperm parameters and histopathological changes relative to the vehicle group (*p* < 0.05). In the vehicle group, antioxidant enzyme activities and the expression of Sertoli cell Vimentin filaments obviously decreased, while lipid peroxidation and AI significantly increased as compared with the control group (*p* < 0.05). Following ICAII treatment, corrective effects on these items towards normal levels were observed. The results suggested that ICAII has beneficial effect on the preservation of spermatogenic function in the STZ-induced diabetic rats. The mechanisms might be related to its improvement of antioxidant enzyme activities, preservation of the protein expression and apical extensions of Vimentin filaments, and anti-apoptosis capability.

## 1. Introduction

Diabetes mellitus (DM) is a chronic progressive metabolic disorder characterized by hyperglycemia that causes severe complications and deleterious effects on various organs in the body. There are growing evidences proving that the excess generation of reactive oxygen species (ROS) in diabetes, which cause oxidative stress, may wholly or in part contribute towards the development of complications in a variety of tissues [1,2,3]. Currently, reproductive dysfunctions in diabetic males are well documented [4,5]. The diabetogenic agent STZ, isolated from Streptomyces achromogenes, is a genotoxic agent and a potential source of oxidative stress. The STZ-induced diabetes rat model is widely used in basic studies to evaluate the effects of DM on male infertility. Shrilatha B *et.al*. have demonstrated that the increased oxidative stress in the STZ-induced diabetic model participates in the development of spermatogenic dysfunction [6].

Keeping the balance between the production of ROS and their catabolism by antioxidants is a critical mechanism in preventing damage from oxidative stress. Therefore, supplementation with antioxidants, such as flavonoids, has been used to prevent the STZ-induced reproductive dysfunctions [7]. Icariin is a flavonol extracted from Herba epimedii, which has been traditionally used in clinical practice for over 2000 years in East Asian countries. Various studies [8,9,10] have demonstrated that icariin has obvious effects on antioxidant defenses and strengthening Yang. ICAII, as the bioactive form of icariin *in vivo*, lacks a glucose moiety at C-7 and is more bioavailable than icariin [11,12]. Recently, we have demonstrated that ICAII has a benefit effect on improving of erectile function in a STZ-induced rat model [13]. Erectile dysfunction (ED) is another causative factor for male infertility under diabetic conditions [14]. Thus, we hypothesized that ICAII could improve the fertility potential in diabetic males through both direct and indirect effects: preservation of normal spermatogenisis through antioxidant effect and improvement of erectile function.

The present study focused on two questions: (1) whether ICAII treatment could prevent spermatogenic dysfunction and improve sperm parameters in the STZ-induced rat model; (2) what is the mechanism behind the correct effect of ICAII on spermatogenic dysfunction in STZ-induced diabetic male rats, if any. If the answer to the first question is YES, ICAII will have good prospects for the prevention and treatment of diabetes-related male infertility.

## 2. Results and Discussion

### 2.1. Results

#### 2.1.1. Blood Glucose Level, Body Weight and Reproductive Organ Weight

**Figure 1:**
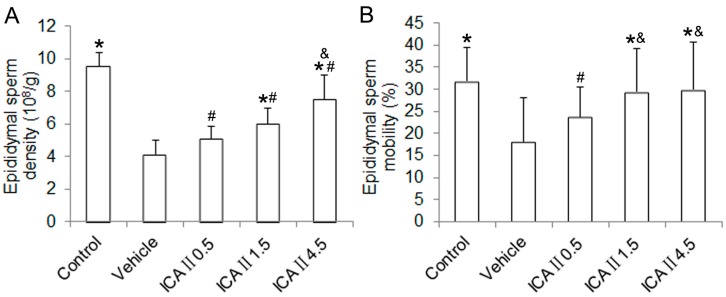
Effect of ICAIItreatment on the epididymal sperm parameters. (**A**) epididymal sperm density; (**B**) epididymal sperm mobility. * *p* < 0.05 compared with the vehicle group; ^#^ *p* < 0.05 compared with the control group; ^&^ *p* < 0.05 compared with the ICAII1.5 group.

The blood glucose level, body weight, reproductive organs weight (testes and epididymis) and sperm parameters of the rats are presented in Table 1. The blood glucose level of the vehicle group was significantly higher than that of the normal control group (*p* < 0.05). In addition, the final body weight and reproductive organ weight of the rats in the vehicle group were significantly lower than those in the normal control group (*p* < 0.05). However, no significant differences in blood glucose level, body weight and reproductive organ weight were found between the vehicle group and ICA II-treated groups. The STZ-induced diabetic rats showed a significantly decreased epididymal sperm density and epididymal sperm mobility as compared with the normal controls. Conversely, diabetic rats treated with ICAII showed a dose-dependent improvement of these two sperm parameters in comparison with the STZ-induced rats (Figure 1).

**Table 1:** Levels of blood glucose, body weight, reproductive organs weight, and epididymal sperm parameters.

Parameter	Control	Vehicle	ICAII0.5	ICAII1.5	ICAII4.5
**Blood glucose (mg·dL^−1^)**	106.85 ± 15.81	423.66 ± 13.73 *	421.32 ± 15.87 *	421.08 ± 15.67 *	419.26 ± 17.85 *
**Initial body weight (g)**	247.33 ± 9.48	246.83 ± 8.13	247.67 ± 12.83	248.17 ± 8.57	247.33 ± 11.29
**Final body weight (g)**	453.83 ± 10.26	345.83 ± 10.57 *	347.33 ± 12.32 *	344.33 ± 9.79 *	346.17 ± 14.27 *
**Testis weight (g)**	3.23 ± 0.13	2.08 ± 0.08 *	2.10 ± 0.06 *	2.07 ± 0.12 *	2.10 ± 0.09 *
**Epididymis weight (g)**	1.46 ± 0.11	1.13 ± 0.15 *	1.11 ± 0.11 *	1.11 ± 0.09 *	1.16 ± 0.20 *

Control: the control group; Vehicle: the STZ-induced diabetic rats received vehicle; ICAII0.5, 1.5 and 4.5: the STZ-induced diabetic rats treated with ICAII (0.5, 1.5 or 4.5 mg/kg/day respectively). * *p* < 0.05 compared with the control group.

**Figure 2:**
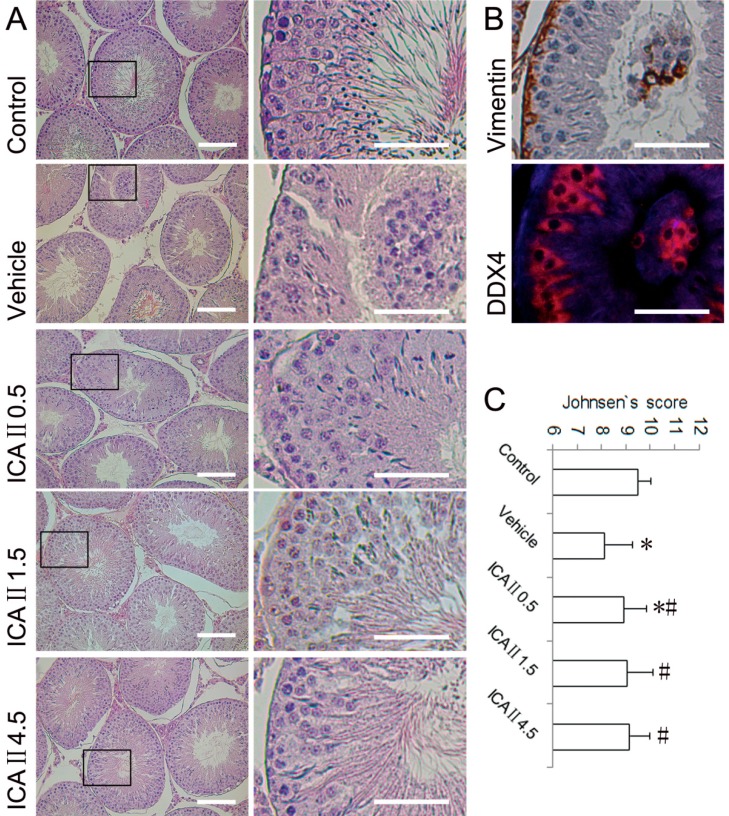
Light microscopy of testicular histopathology in different groups. (**A**) Representative micrographs (haematoxylin and eosin staining) of the testes. Boxed areas in the 200× graphs are shown in the corresponding graphs on the right panels; (**B**) **Top**: Some detached cells in the lumen of seminiferous tubule are confirmed to be Vimentin (a sertoli marker) positive cell (immunohistochemical staining), 400×; **Bottom**: Some deciduous cells in the lumen were DDX4 (a germ cell marker, red) positive cells (immunofluorescent staining), 1000×; (**C**) Johnsen’s testicular score. * *p* < 0.05 compared with the normal control group; ^#^ *p* < 0.05 compared with the vehicle group. Scale bar = 50 μm.

#### 2.1.2. Histopathological Changes

Testicular histological sections and Johnsen’s scores of the five groups are presented in Figure 2. The normal controls showed a presence of regular testicular architecture and seminiferous tubular morphology with complete spermatogenic cell series. A significant and varying degree of histopathological changes was observed in the testes of STZ-induced rats at week 4. The typical pathological lesions included disorganisation and desquamation of germinal cells. The deciduous cells in the seminiferous tubular lumen were further confirmed to be Sertoli cells (Vimentin-positive) and germ cells (DDX4-positive). It was also found that the mean Johnsen’s score was significantly decreased in the vehicle group compared with the control group. Oral administration of ICAII to STZ induced diabetic rats caused a marked amelioration in testicular histopathological changes. Partial but significant recoveries of Johnsen’s score were observed in all ICAII treated groups. Although the Johnsen’s scores increased in a dose-dependent manner when treated with ICAII, the differences were not statistically significant among the ICAII-treated groups.

**Figure 3:**
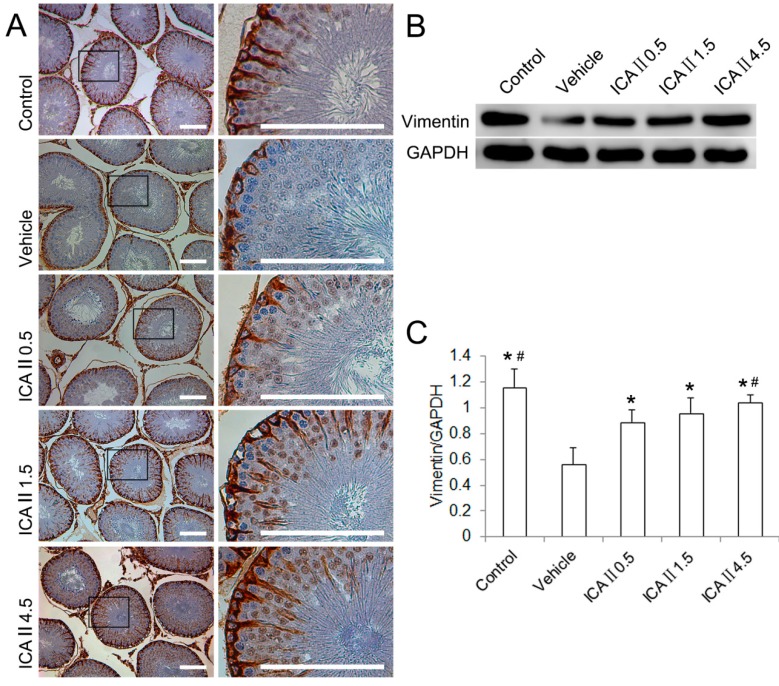
The changes of Sertoli cell Vimentin filaments. (**A**) Distribution and appearance of Sertoli cell Vimentin filaments (immunohistochemical staining). The Vimentin filaments in the control group have a characteristic “spoke-like” appearance; in the vehicle group, the Vimentin filaments lose their apical extensions and mainly concentrate surrounding the nucleus; ICAII (0.5, 1.5 or 4.5 mg/kg/day, respectively) treatments preserve the apical extensions of the Vimentin filaments in a dose-dependent manner. Boxed areas in the 100× graphs are shown in the corresponding graphs on the right panels; (**B**) The protein expression of the Vimentin filaments (western blot); (**C**) Semi-quantitative analysis of Vimentin filaments protein expression in testes with WB. * *p* < 0.05 compared with the vehicle group; ^#^ *p* < 0.05 compared with the ICAII0.5 group. Scale bar = 100 μm.

#### 2.1.3. Disruption of Sertoli Cell Vimentin Filaments

Vimentin, an important component of the Sertoli cell cytoskeleton, is responsible for the anchoring of germ cells to the seminiferous epithelium. The distribution and expression of Sertoli cell Vimentin filaments were detected by immunohistochemistry (IHC) and western blot (WB), respectively (Figure 3). In normal controls, Vimentin filaments radiated from the perinuclear region of Sertoli cell toward the lumen of the tubules with apical “spoke-like” pattern. However, the Sertoli cell Vimentin filaments concentrated surrounding the nucleus and lost the appearance of apical extensions in the seminiferous epithelium of STZ-induced diabetes rats. Consequently, spermatogenic cells dissociated from the Sertoli cells and sloughed into the lumen of the tubules. WB showed the expression of Vimentin filaments was significantly decreased in the vehicle group as compared with the control group. ICAII treatment preserved the apical extensions of the Sertoli cell Vimentin filaments. The expression of the Sertoli cell Vimentin filaments in all ICAII treated groups was significantly higher than that in the vehicle group. In addition, the expression of Vimentin filaments was significantly different between the ICAII 0.5 group and the ICAII 4.5 group, whereas the ICAII 1.5 group did not differ significantly from the lowest and highest doses.

#### 2.1.4. Lipid Peroxidation and Antioxidant Enzyme Activity

**Figure 4:**
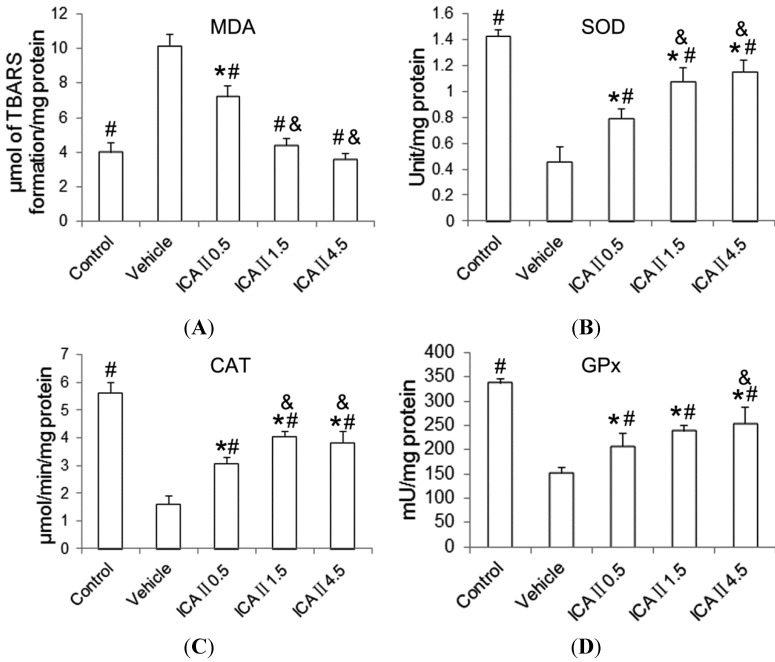
Effects of ICAII treatment on lipid peroxidation (MDA) and antioxidant enzyme activities (SOD, CAT and GPx). (**A**) MDA, Malondialdehyde; (**B**) SOD, superoxide dismutase; (**C**) CAT, catalase; (**D**) GPx, glutathione peroxidase. Values are presented mean± standard deviation. * *p* < 0.05 compared with the control group; ^#^ *p* < 0.05 compared with the vehicle group; ^&^ *p* < 0.05 compared with the ICAII0.5 group.

Malondialdehyde (MDA) levels of the testicular tissue increased significantly (*p* < 0.05) in the STZ-induced diabetic rats as compared with the control group. Oral administration of ICAII to diabetic rats resulted in a significant decrease of MDA to normal level in a dose-dependent manner. The level of MDA in the normal control group was not significantly different from the moderate or the highest dose. Figure 4 represents the levels of superoxide dismutase (SOD), catalase (CAT) and glutathione peroxidase (GPx) in testicular tissues of control and experimental rats. Significant decreases in the levels of SOD, CAT and GPx were observed in the STZ-induced diabetic rats when compared with the normal controls. The activities of these endogenous antioxidants in the STZ-induced rats were significantly elevated towards the control level, in a dose-dependent manner, following gavage feeding of ICAII (0.5, 1.5 or 4.5 mg/kg/day). However, there were still significant differences in the levels of SOD, CAT and GPx activity between the normal control and the ICAII4.5 group. The inconsistency between MDA and antioxidant enzymes indicated that the antioxidant capacity of ICAII itself may also play an important role in the maintenance of testicular oxidation-reduction state.

#### 2.1.5. Analysis of Apoptotic Index (AI)

**Figure 5:**
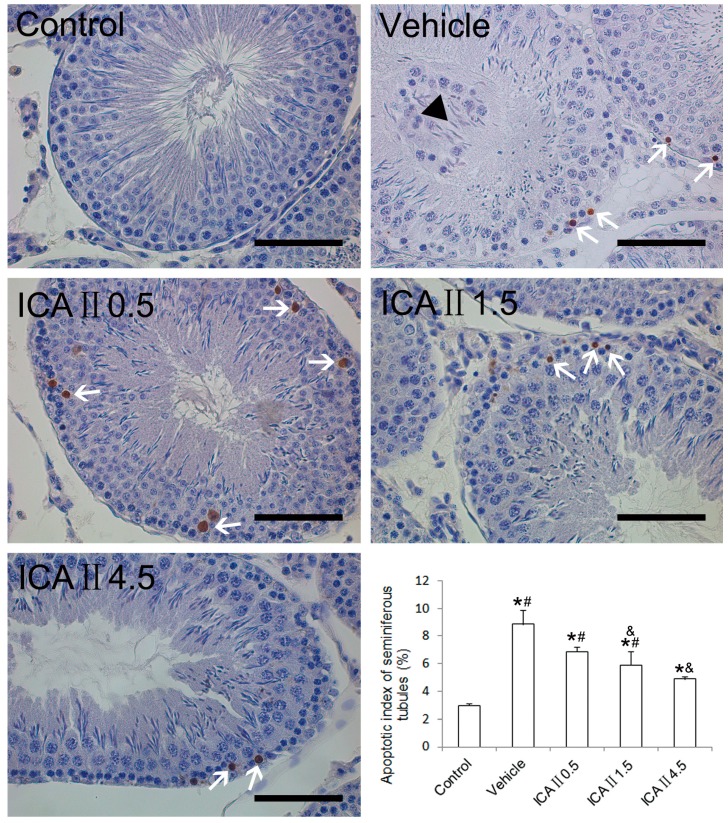
Representative TUNEL staining of the seminiferous tubules. Brown-stained cells are undergoing apoptosis (white arrows). The black triangle represents the deciduous cells in the lumen of seminiferous tubule. Seminiferous tubules containing two or more TUNEL-positive cells are positive tubules of apoptosis. AI is calculated as the ratio of the positive tubules to total tubules. Quantitative data of AI are shown in the bar chart on the lower right corner. 400×. * *p* < 0.05 compared with the control group; ^&^ *p*< 0.05 compared with the vehicle group; ^#^ *p* < 0.05 compared with the ICAII4.5 group. Scale bar = 50 μm.

The rate of terminal transferase-mediated dUTP-biotin nick end-labeling (TUNEL) positive cells was generally very low in normal controls (Figure 5). In the present study, we found that the AI value in the STZ-induced diabetic rats (about 8.82%) was significantly higher than that in the normal controls (about 2.94%). However, the deleterious effect was partially but significantly reversed following gavage feeding of ICAII (0.5, 1.5 or 4.5 mg/kg/day). The value of AI decreased to an average of 6.84% in the ICAII0.5 group, 5.87% in the ICAII1.5 group and 4.87% in the ICAII4.5 group. However, the AI value in the ICAII4.5 group was still higher than that in the normal control group. These results suggested that oxidative stress may not be the only mechanism by which resulted in cell apoptosis in the seminiferous epithelium.

### 2.2. Discussion

It is well known that oxidative stress plays an important role in the pathogenesis and development of complications of two types of DM. Currently, numerous epidemiological and experimental evidences have demonstrated that there is a potential relationship between testicular oxidative damage during DM and male infertility [15,16]. In the rat model of diabetes, induced by STZ, earlier study found that oxidative stress in the testicular milieu contributed to the development of testicular dysfunctions [17]. In addition, STZ-induced diabetic rat is also widely used as a model for the study of ED, which is another causative factor for male infertility. In our previous study, we found that ICAII could improve erectile function of the STZ-induced rat model by repairing penile pathological changes in a dose-dependent manner [13].

STZ enters the beta cells via a glucose transporter (GLUT2) and causes DNA fragmentation which induces the loss of pancreatic beta cells and subsequent hyperglycemia. The underlying mechanism is mainly due to its potent DNA alkylating [18]. In addition, STZ was found to generate ROS, which also contribute to DNA damage [19]. ICAII has wide protective effects in diseases and can increase oxidative stress tolerance [12]. Our previous results [13] showed that the high dose ICAII (10 mg/kg/day) had a trivial hypoglycemic effect, which might be related with its antioxygenation in the pancreas. However, ICA II treatments had no influence on the blood glucose level in the present dosage range (less than or equal to 4.5 mg/kg/day). The hypoglycemic effect and its mechanisms of high dose ICAII should be further studied in the future.

Endogenous antioxidant defense system which protect against reactive oxygen species, includes both enzymatic (SOD, CAT and GPx) and non-enzymatic (glutathione (GSH), Vitamins A, C and E) pathways [6]. In the present study, we found that lipid peroxidation level in the testicular tissue significantly increased, while the activities of SOD, CAT and GPx significantly decreased in the STZ-induced diabetic rats as compared with the normal controls. Oral administration of ICAII to diabetic rats resulted in a significant recovery of the thiobarbituric acid (TBA) reactive substances (TBARS) towards the normal level in a dose-dependent manner. Especially in the moderate (1.5 mg/kg/day) and high dose groups (4.5 mg/kg/day), the levels of TBARS were almost equivalent to that in the normal control group.

Unlike the lipid peroxidation level, the activities of the three antioxidant enzymes mentioned above could be significantly but only partially reversed following ICAII treatment. Even in the high dose group, the three enzymatic activities were still significantly lower than those in the normal control group. The inconsistency between MDA and antioxidant enzymes raises a logical question—whether ICAII treatment suppressed lipid peroxidation through an additional non-enzymatic pathway. While it is difficult to precisely answer this question based on the data obtained in the present study, it can be reasonably postulated that the antioxidant capacity of ICAII itself may also play an important role in the maintenance of testicular oxidation-reduction state based on previous researches.

Sertoli cell, a somatic cell in the seminiferous epithelium, is known to provide the appropriate hormonal, nutritional and physical support for spermatogenic cell development and maturation [20]. Vimentin filaments in Sertoli cell play a major role in these processes, and they are responsible for anchoring spermatogenic cells to the underlying Sertoli cells. As Sertoli cells are involved in the regulation of spermatogenesis, any metabolic alteration in these cells derived from DM may be responsible for spermatogenesis disruption [21]. Previous studies have demonstrated that collapse of Vimentin filaments causes the separation of spermatogenic cells from seminiferous epithelium and finally undergoing apoptosis [22,23]. In this study, IHC staining showed that STZ treatment altered the distribution of Vimentin filaments in Sertoli cells, characterized by loss of their apical extensions and collapse around Sertoli cell nuclei. WB results further confirmed a noticeable reduction in the expression of Vimentin filament protein in the STZ-induced diabetic rats.

Interestingly, ICAII treatment preserved the apical extensions and protein expression of Sertoli cell Vimentin filaments in a dose-independent manner. In addition, the expression of Vimentin filaments in the ICAII 4.5 group did not differ from that in the normal control group. As expected, the apoptotic index of STZ-induced diabetic rats significantly increased as compared with that of the normal controls. Although the AI values of diabetic rat significantly decreased following daily ICAII administration, the best treatment effect in the ICAII 4.5 group was still significantly higher than that in the normal control group. These results suggested that oxidative stress or Vimentin filament collapse may not be the only mechanisms by which resulted in seminiferous cell apoptosis. In this study, our results showed that ICAII treatment could not regulate the blood sugar level, so we speculated that persistent hyperglycemia may also be a direct cause of cell apoptosis.

## 3. Experimental Section

### 3.1. Animals

A total of 50 male Sprague-Dawley (SD) rats (12 weeks old) were obtained from the Animal Breeding Center at the Peking University Health Science Center. The experiments were approved by the institutional animal care and use subcommittee of our university. All animals were maintained under the controlled conditions of temperature (23 ± 2 °C) and light (12 h light/dark cycle) with standard diet and water. Ten SD rats were randomly selected as age-matched controls (intraperitoneal injection of citrate buffer) and received vehicle treatment. The remaining 40 rats received an intraperitoneal injection of STZ in citrate buffer (55 mg/kg), were randomly divided into four equal groups, and treated with daily solvent (vehicle group) or ICAII (0.5, 1.5 or 4.5 mg/kg) by gavage feeding. All rats were fasted for 16 hours prior to STZ injection. The treatment was continued for 4 weeks, followed by a wash-out period of 72 h, and then all rats were sacrificed. Genital glands (caudal epididymis and testes) were dissected out and spermatozoa were collected from the epididymis for analysis. Haematoxylin and eosin (HE) staining, IHC, WB and TUNEL assay were conducted. Slides were photographed and recorded using a Leica DFC 425 C digital microscope camera system (Leica, Heidelberg, Germany). Computerized histomorphometric analysis was performed using Image-Pro Plus 6.0 software (Media Cybernetics, Bethesda, MD, USA).

### 3.2. Plasma Glucose, Body and Reproductive Organ Weights

Blood glucose levels were monitored at a regular interval throughout the study using a blood glucose analyzer (B. Braun, Melsungen, Germany). Body weights of all animals were weighed twice at the beginning and end of the study. Immediately after sacrifice, testes and caudal epididymis were excised and their weights were recorded.

### 3.3. Evaluation of Epididymal Sperm Density and Epididymal Sperm Motility

Sperm analysis was conducted as our previous description [24]. The bilateral caudal epididymis was dissected out and spermatozoa were collected in 2 mL medium (Hams F10) containing 0.5% bovine serum albumin. After 5 min incubation at 37 °C, the epididymal sperm count was determined using the standard hemocytometric method and epididymal sperm motility was analyzed microscopically in 10 fields under a light microscope (Leica, Heidelberg, Germany) using a 40× objective according to the World Health Organization recommended method. Epididymal sperm density was calculated as the ratio of the epididymal sperm count to caudal epididymis weight.

### 3.4. Measurement of Lipid Peroxidation and Antioxidant Enzyme Assays

Lipid peroxidation was assessed in tissue cytosol by measuring TBARS and quantified as MDA levels. Briefly, the supernatant fraction was mixed with TBA reagent consisting of 0.375% TBA and 15% trichloroacetic acid (TCA) in 0.25 mM hydrochloric acid. The reaction mixtures were placed in boiling water, and then the absorbance of the supernatant was measured at 535 nm. MDA levels were expressed as mmol/mg protein.

SOD activity (S0101, Beyotime Biotechnology, Shanghai, China), CAT activity (S0051, Beyotime Biotechnology, Shanghai, China) and GPx activity (S0058, Beyotime Biotechnology, Shanghai, China) were all determined using commercial kits following the manufacturer’s instructions. Briefly, SOD activity was assayed by monitoring the inhibition of 2-(2-methoxy-4-nitrophenyl)-3-(4-nitrophenyl)-5-(2,4-disulfophenyl)-2*H*-tetrazolium (WST-8) formazan, using xanthine–xanthine oxidase as the source of superoxide anion, at 450 nm spectrophotometrically. CAT activity was measured by detecting the colored product *N*-(4-antipyryl)-3-chloro-5-sulfonate-*p*-benzoquinonemonoimine at 520 nm spectrophotometrically. GPx activity was determined as manufacturer’s protocol at 340 nm spectrophotometrically. The bicinchoninic acid (BCA) assay was used for protein quantitation.

### 3.5. HE Staining, IHC and TUNEL

The testicular tissues were harvested and fixed in Bouin’s solution for a period of 6 h and then transferred to 70% ethanol until processing. The fixed tissues were dehydrated in alcohol gradient, cleared in xylene, and embedded in paraffin. Sections with a thickness of 4 μm were cut using a rotor microtome. The paraffin sections were then dewaxed in xylene for 20 min, rehydrated in serial graded ethanol and then used for HE staining, IHC and TUNEL assay.

For HE staining, the histopathological changes in testicular tissue were evaluated by Johnsen’s testicular score system. Thirty cross-sectioned tubules in each group were randomly selected for evaluation, and a score between 1 (very poor) and 10 (excellent) was given to each tubule according to Johnsen’s criteria [25].

The endogenous peroxidase was inactivated with 0.3% hydrogen peroxide at room temperature for 30 min. After blocking with PBS containing 0.1% Triton X-100 and 5% goat serum, the slides were subsequently incubated with antibodies to mouse anti-Vimentin (a sertoli cell marker, 1:50, Santa Cruz, CA, USA) and rabbit anti-DDX4 (a germ cell marker, 1:400, Abcam, Burlingame, CA, USA), and stored overnight at 4 °C in a humidified chamber. The sections for immunohistochemical staining were incubated with the MaxVision Horse Reddish Peroxidase (HRP)-Polymer anti-rabbit immunohistochemistry kit (Maxim, Fuzhou, China) and were developed color with diaminobenzidine (DAB). The sections for immunofluorescent staining were subsequently washed with PBS and were incubated at room temperature for 1.5 h with AlexaFluor-594 conjugated secondary antibody (Sigma Aldrich, St. Louis, MO, USA).

The TUNEL assay was performed according to the manufacturer’s instructions (KGA7032, KeyGEN BioTECH, Najing, China). Briefly, the sections were treated with Proteinase K (20 mg/L) for 30 min (KeyGEN BioTECH, China) at 37 °C. 50 μL reaction mixture containing 45 μL equilibration buffer, 4 μL TdT enzyme and 1 μL biotin-11-dUTP was then added to each sample. After 60 min incubation at 37 °C, the sections were washed with PBS three times and incubated with the streptavidin-HRP for 30 min at 37 °C. The sections were then treated with 3,3-diaminobenzidine (DAB) for 5–8 min, counterstained with hematoxylin. The seminiferous tubules (in 30 randomly selected fields) containing two or more TUNEL-positive cells were counted as positive. The apoptosis index (AI) in each group was calculated as the ratio of the positive tubules of apoptosis to total tubules in a cross section.

### 3.6. Western Blotting

The cellular lysates from testicular tissue containing 20 μg protein were electrophoresed in sodium dodecyl sulfate-polyacrylamide gel electrophoresis and then transferred to a polyvinylidene fluoride membrane (Millipore Corp, Bedford, Massachusetts, Bedford, MA, USA). Primary antibodies were mouse anti-Vimentin (Sertoli cell marker, 1:50, Santa Cruz, CA, USA), and rabbit anti-reduced glyceraldehyde-phosphate dehydrogenase (GAPDH, 1:2000, Santa Cruz, CA, USA). After hybridization of secondary antibodies, the resulting images were analyzed with ChemiImager 4000 (Alpha Innotech Corporation, San Leandro, CA, USA).

### 3.7. Statistical Analysis

All data are expressed as mean ± SD (the standard deviation). Statistical Package for social Sciences software package version 17.0 (SPSS Inc., Chicago, IL, USA) was used for all statistical analyses. Semi-quantitative analysis was performed using Image-pro plus software (Bethesda, MD, USA). Multiple groups were compared using one-way analysis of variance followed by the Tukey HSD *post hoc* comparisons, and *p* < 0.05 was considered significant.

## 4. Conclusions

In summary, the above results suggested that ICAII treatment has beneficial effects on the preservation of normal testicular spermatogenic function in STZ-induced diabetic rats. The underlying mechanism might be related to improvement of endogenous antioxidant enzyme activities (indirect effect), preservation of Vimentin filaments apical extensions, anti-apoptosis and its non-enzymatic antioxidant capability (direct effect). Based on our results, it can be reasonably postulated that ICAII could provide two pathways to improve the fertility potential in diabetic males: preservation of normal spermatogenisis and improvement of erectile function.
